# Editorial: Epigenetic and genetic mechanisms underlying cardiovascular diseases and neurodevelopmental disorders

**DOI:** 10.3389/fgene.2024.1401354

**Published:** 2024-04-03

**Authors:** Peng Zhang, Lingshan Gou, Dharmani Devi Murugan, Hongsong Zhang

**Affiliations:** ^1^ Shenzhen Key Laboratory of ENT, Institute of ENT and Longgang ENT Hospital, Shenzhen, China; ^2^ Center for Genetic Medicine, Xuzhou Maternity and Child Healthcare Hospital Affiliated to Xuzhou Medical University, Xuzhou, China; ^3^ Department of Pharmacology, Faculty of Medicine, Universiti of Malaya, Kuala Lumpur, Malaysia; ^4^ Department of Cardiology, Nanjing First Hospital, Nanjing Medical University, Nanjing, Jiangsu, China

**Keywords:** genetics, cardiovascular disease, neurodevelopmental disorder, epigenetics, coronary artery disease

Cardiovascular diseases (CVD) and neurodevelopmental disorders (NDD) represent significant health challenges, posing severe threats to human health and quality of life. Emerging as the foremost cause of mortality and disability globally, CVD is intricately linked with a variety of factors such as chronic inflammation and genetic predispositions (Dzau and Hodgkinson, 2024). Similarly, NDD, characterized by deficits in cognitive functions, social interactions, and learning capabilities (Olson et al., 2024), demands deeper understanding of their genetic and epigenetic underpinnings. The complexities of these diseases stem from a multifaceted etiology involving DNA variants, epigenetic modifications, and environmental influences, a narrative supported by research from Srour and Shevell (2014), Homberg et al. (2016), and Hartiala et al. (2021).

Chronic inflammation plays a pivotal role in CVD, such as acute myocardial infarction (AMI) and coronary artery disease (CAD) (Liu C. et al., 2022; Lopez-Candales et al., 2017). Macrophage migration inhibitory factor (MIF) is an important pro-inflammatory cytokine implicated in the pathogenesis of CVD (Zernecke et al., 2008). *MIF* gene, located at 22q11.2, features polymorphisms such as −173 G/C polymorphism (rs755622), and −974 CATT tetranucleotide repeat (rs5844572), which may influence gene transcription and inflammatory processes. Fouda et al. conducted a meta-analysis of the MIF -173G/C variant’s impact on CVD risk in 9,047 participants, revealing its association with increased CVD risk in specific populations, highlighting the genetic underpinnings of inflammation in CVD.

AMI is a life-threatening disease involving thrombosis, fibrinolysis, inflammation, and lipid metabolism. Jeon et al. identified six early-onset AMI-associated variants, notably rs12639023 as a prognostic marker for cardiac mortality. This genetic perspective is crucial for understanding AMI’s complex pathogenesis. Despite extensive research, the understanding of epigenetic alterations in endothelial cells and their implication in the etiopathology of AMI remains incomplete. Tang et al. addressed this gap by investigating DNA methylation alterations and altered gene expression profile in endothelial cells exposed to oxygen-glucose deprivation. Their findings shed light on oxygen-glucose deprivation-specific genes implicated in coronary endothelial cell injury during AMI.

CAD comprised a broad spectrum of clinical syndromes induced by inadequate blood flow to the myocardium, primarily attributed to subintimal atheroma deposition resulting in arterial stenosis, occlusion and wall thickening (Knuuti et al., 2020). MicroRNA (miRNA) orchestrate multiple crucial processes such as angiogenesis, cell proliferation, differentiation, migration, and apoptosis within the circulatory system. MiRNAs have the potential in facilitating early detection, assessing disease severity, and predicting outcomes in CAD. Lv et al. reported significantly elevated miR-183-5p levels in CAD patients across varying disease severities compared to non-CAD individuals. These results underscore the potential of serum miR-183-5p levels as a predictive biomarker for CAD presence and severity, offering valuable insights into disease progression and prognosis.

Sick Sinus Syndrome (SSS), atrial fibrillation (AF), and pulmonary arterial hypertension (PAH), also represent significant challenges within the realm of CVD. SSS arises from the sinus node dysfunction or impaired electrical impulse conduction, culminating in sinus bradycardia, sinus block, or sinus arrest (De Ponti et al., 2018). Genetic mutations have been linked to familial SSS. Liang et al., identified heterozygous mutations of *SCN5A* gene in three young familial SSS females, including novel mutation sites not previously reported in Asian patients. Advancements in genetic research have elucidated the genetic substrates of AF, with early-onset AF potentially indicating genetic atrial myopathy (Kany et al., 2021). Silva Cunha et al. reported a young AF patient with extensive atrial fibrosis and extensive areas of low voltage. Genetic analysis unveiled a homozygous pathogenic variant in *NPPA*, which was parentally inherited. This case underscores the role of genetic predispositions, particularly NPPA mutations, in AF pathogenesis and atrial fibrosis development.

Epigenetic modifications and abnormal immune microenvironment are key factors in PAH pathogenesis (Kim et al., 2015; Liu J. et al., 2022). N6-methyladenosine (m6A) RNA modification, a critical epitranscriptomic mechanism, regulates RNA biology. Gao et al. analyzed the data from the GSE117261 dataset, identifying differential expression of genes (DEGs) and m6A regulators in idiopathic PAH (IPAH) samples. Functional and pathway enrichment analyses incorporating 77 DEGs further underscored aberrant immune activity implicated in IPAH pathogenesis. Notably, histone lactylation, a novel post-translational modification, also assumes significance in PAH. Zhao et al. reviewed the role of histone lactylation in PAH, and its effects on N6-methyladenosine (m6A) and immune microenvironment. These insights offer novel perspectives into PAH diagnosis and pathogenesis.

Benign familial infantile epilepsy (BFIE), late-stage mild cognitive impairment (LMCI) and Alzheimer’s disease (AD) represent critical areas of concern within neurodevelopmental and neurodegenerative disorders (Vigevano, 2005; Dakterzada et al., 2023; Sun et al., 2023). BFIE is a familial epileptic syndrome, characterized with focal seizures that may evolve to secondary generalized tonic-clonic seizures. *PRRT2* gene, encoding the proline-rich transmembrane protein 2, is a major causative gene for BFIE (Massimino et al., 2023). Gu et al. reported seven cases of BFIE effectively managed with anti-seizure medication, all stemming from pathogenic PRRT2 variants. Notably, among these variants, the frameshift variant c.397delG was novel, highlighting the importance of whole-exome sequencing in BFIE diagnosis.

In the realm of neurodegenerative diseases, the transition from late-stage mild cognitive impairment (LMCI) to AD poses a significant risk for cognitive decline (Tábuas-Pereira et al., 2016). Zhang et al. explored the association of peripheral blood methylation profiles between individuals experiencing cognitive aging and those diagnosed with LMCI. Abnormal methylation signal intensities for some genes have been identified to be related to an enhanced susceptibility to AD. These findings illuminate the complex interplay between DNA methylation patterns and gene expression regulation in the context of cognitive impairment and Alzheimer’s disease progression, providing potential avenues for further exploration in diagnostic and therapeutic interventions.

In summary, the understanding of the pathogenic mechanisms underlying CVD and NDD remain incomplete due to their complex etiologies. However, the studies featured in the current Research Topic have made significant strides in unraveling these complexities. Primarily focusing on epigenetic and genetic factors, the investigations delved into the intricate etiologies and potential biomarkers crucial for diagnosing these conditions. The findings presented in these studies represent valuable contributions to the existing body of knowledge regarding the origins, progression, and diagnostic strategies for these diseases.

## References

[B1] Dakterzada F. Jové M. Huerto R. Carnes A. Sol J. Pamplona R. (2023). Changes in plasma neutral and ether-linked lipids are associated with the pathology and progression of Alzheimer’s disease. Aging Dis. 14 (5), 1728–1738. 10.14336/AD.2023.0221 37196122 PMC10529749

[B2] De Ponti R. Marazzato J. Bagliani G. Leonelli F. M. Padeletti L. (2018). Sick sinus syndrome. Card. Electrophysiol. Clin. 10, 183–195. 10.1016/j.ccep.2018.02.002 29784479

[B3] Dzau V. J. Hodgkinson C. P. (2024). RNA therapeutics for the cardiovascular system. Circulation 149 (9), 707–716. 10.1161/CIRCULATIONAHA.123.067373 38408142

[B4] Hartiala J. A. Hilser J. R. Biswas S. Lusis A. J. Allayee H. (2021). Gene-environment interactions for cardiovascular disease. Curr. Atheroscler. Rep. 23 (12), 75. 10.1007/s11883-021-00974-9 34648097 PMC8903169

[B5] Homberg J. R. Kyzar E. J. Scattoni M. L. Norton W. H. Pittman J. Gaikwad S. (2016). Genetic and environmental modulation of neurodevelopmental disorders: translational insights from labs to beds. Brain. Res. Bull. 125, 79–91. 10.1016/j.brainresbull.2016.04.015 27113433

[B6] Kany S. Reissmann B. Metzner A. Kirchhof P. Darbar D. Schnabel R. B. (2021). Genetics of atrial fibrillation-practical applications for clinical management: if not now, when and how? Cardiovasc. Res. 117 (7), 1718–1731. 10.1093/cvr/cvab153 33982075 PMC8208749

[B7] Kim J. D. Lee A. Choi J. Park Y. Kang H. Chang W. (2015). Epigenetic modulation as a therapeutic approach for pulmonary arterial hypertension. Exp. Mol. Med. 47 (7), e175. 10.1038/emm.2015.45 26228095 PMC4525299

[B8] Knuuti J. Wijns W. Saraste A. Capodanno D. Barbato E. Funck-Brentano C. (2020). 2019 ESC Guidelines for the diagnosis and management of chronic coronary syndromes. Eur. Heart. J. 41 (3), 407–477. 10.1093/eurheartj/ehz425 31504439

[B9] Liu C. Sun Z. Shali S. Mei Z. Chang S. Mo H. (2022a). The gut microbiome and microbial metabolites in acute myocardial infarction. J. Genet. Genomics. 49 (6), 569–578. 10.1016/j.jgg.2021.12.007 34974193

[B10] Liu J. Yang P. Tian H. Zhen K. McCabe C. Zhao L. (2022b). Right ventricle remodeling in chronic thromboembolic pulmonary hypertension. J. Transl. Int. Med. 10 (2), 125–133. 10.2478/jtim-2022-0027 35959451 PMC9328037

[B18] Lopez-Candales A. Hernández Burgos P. M. Hernandez-Suarez D. F. Harris D. (2017). Linking Chronic Inflammation with Cardiovascular Disease: From Normal Aging to the Metabolic Syndrome. J. Nat. Sci. 3 (4), e341. 28670620 PMC5488800

[B11] Massimino C. R. Portale L. Sapuppo A. Pizzo F. Sciuto L. Romano C. (2023). RRT2 related epilepsies: a gene review. J. Pediatr. Neurol. 21 (04), 264–272. 10.1055/s-0041-1728683

[B12] Olson L. Bishop S. Thurm A. (2024). Differential diagnosis of autism and other neurodevelopmental disorders. Pediatr. Clin. North. Am. 71 (2), 157–177. 10.1016/j.pcl.2023.12.004 38423714 PMC10904885

[B13] Srour M. Shevell M. (2014). Genetics and the investigation of developmental delay/intellectual disability. Arch. Dis. Child. 99 (4), 386–389. 10.1136/archdischild-2013-304063 24344174

[B14] Sun Y. Hefu Z. Li B. Lifang W. Zhijie S. Zhou L. (2023). Plasma extracellular vesicle microRNA analysis of Alzheimer’s disease reveals dysfunction of a neural correlation network. Research 6, 0114. 10.34133/research.0114 37223486 PMC10202186

[B15] Tábuas-Pereira M. Baldeiras I. Duro D. Santiago B. Ribeiro M. H. Leitão M. J. (2016). Prognosis of early-onset vs late-onset mild cognitive impairment: comparison of conversion rates and its predictors. Geriatrics 1 (2), 11. 10.3390/geriatrics1020011 31022805 PMC6371125

[B16] Vigevano F. (2005). Benign familial infantile seizures. Brain Dev. 27 (3), 172–177. 10.1016/j.braindev.2003.12.012 15737697

[B17] Zernecke A. Bernhagen J. Weber C. (2008). Macrophage migration inhibitory factor in cardiovascular disease. Circulation 117 (12), 1594–1602. 10.1161/CIRCULATIONAHA.107.729125 18362243

